# Re-visiting protein-centric two-tier classification of existing DNA-protein complexes

**DOI:** 10.1186/1471-2105-13-165

**Published:** 2012-07-16

**Authors:** Sony Malhotra, Ramanathan Sowdhamini

**Affiliations:** 1 National Centre for Biological Sciences (TIFR), UAS-GKVK Campus, Bellary Road, Bangalore, 560 065, India

**Keywords:** DNA, Classification, DNA-protein interactions, Genome-wide survey, Sequence searches

## Abstract

**Background:**

Precise DNA-protein interactions play most important and vital role in maintaining the normal physiological functioning of the cell, as it controls many high fidelity cellular processes. Detailed study of the nature of these interactions has paved the way for understanding the mechanisms behind the biological processes in which they are involved. Earlier in 2000, a systematic classification of DNA-protein complexes based on the structural analysis of the proteins was proposed at two tiers, namely groups and families. With the advancement in the number and resolution of structures of DNA-protein complexes deposited in the Protein Data Bank, it is important to revisit the existing classification.

**Results:**

On the basis of the sequence analysis of DNA binding proteins, we have built upon the protein centric, two-tier classification of DNA-protein complexes by adding new members to existing families and making new families and groups. While classifying the new complexes, we also realised the emergence of new groups and families. The new group observed was where β-propeller was seen to interact with DNA. There were 34 SCOP folds which were observed to be present in the complexes of both old and new classifications, whereas 28 folds are present exclusively in the new complexes. Some new families noticed were NarL transcription factor, Z-α DNA binding proteins, Forkhead transcription factor, AP2 protein, Methyl CpG binding protein *etc*.

**Conclusions:**

Our results suggest that with the increasing number of availability of DNA-protein complexes in Protein Data Bank, the number of families in the classification increased by approximately three fold. The folds present exclusively in newly classified complexes is suggestive of inclusion of proteins with new function in new classification, the most populated of which are the folds responsible for DNA damage repair. The proposed re-visited classification can be used to perform genome-wide surveys in the genomes of interest for the presence of DNA-binding proteins. Further analysis of these complexes can aid in developing algorithms for identifying DNA-binding proteins and their family members from mere sequence information.

## Background

The driving forces for the cell to survive and regulate its various processes are the specific interactions between macromolecules. Protein-nucleic acid interactions are important for many high fidelity cellular processes. Both of these macromolecules are known to be involved in various important mechanisms and processes of systems biology- replication, transcription, translation, recombination, DNA-repair, DNA packaging etc. Therefore, DNA-binding proteins serve as the key players in maintaining cell viability and proliferation.

Also, DNA-binding proteins constitute both eukaryotic and prokaryotic proteomes. The interplay between DNA and proteins is most fundamental interaction in biology and also has implications in the field of medicine, pharmacology and biotechnology. The diverse function of DNA-binding proteins is accompanied by the diversity in their sequences and structures.

Therefore, to elucidate and understand the mechanism of any of the biological processes involving DNA-binding proteins, it is necessary and useful to study the nature of these nucleic acid-protein complexes formed in order to accomplish the specific function [1]. There have been many earlier attempts to study the nature of contacts between DNA and protein, example, H-bond [2,3], and water mediated interactions [4].

In the past, apart from the concern in understanding the interactions between the two macromolecules, interest had also been focused on classifying DNA-protein complexes. The classification based on the structures of DNA-binding domains was first proposed by Harrison in 1991 [5]. Luscombe and coworkers (2000) classified the DNA-protein complexes into 8 groups and 54 families using the structures of DNA binding domain of the protein and on the basis of similarities of overall protein folds, the complexes were classified into different groups. In this existing classification, each group of proteins exhibit similar DNA binding mode, but proteins in some groups differ in terms of structure, mode of interaction and wide range of recognition sequence [1]. Subsequently, in 2002, there was a classification which was based on the analysis of the structural domains interacting with DNA and then clustering these domains was based on structural similarity [6]. Later, in 2006, there was an attempt towards classifying DNA-protein complexes, using descriptors characterizing DNA-protein interactions like number of atomic contacts at major and minor groove, buried surface area at the interface *etc.*[7].

All the approaches of classification, mentioned above, were protein-centric in nature which implies that the classification was based on the features of the protein partner of the complex. However, in 2006, completely new viewpoint of classification was proposed by Sen *et al.* which was DNA-centric in nature and hence based on the features of nucleic acid part of the complex. They made an attempt to classify these complexes based on a clustering approach that incorporates most of the key structural parameters involved in recognition process [8].

In the present study, we have made an attempt towards protein-centric classification of DNA-protein complexes. To study the nature of these complexes, it is important to understand the structure of the DNA-binding domains present in proteins, namely from the Protein Structure Data Bank (PDB) [9]. With the advancement in number and the resolution of structures of DNA-protein complexes, it became important to revisit the existing classification. The classification we propose is based more on sequence similarity rather than structural alignments. Sequence-based approaches towards understanding DNA-binding proteins will gear the developed classification scheme and search algorithms to search effectively in whole genomes, where mere sequence information is available. Re-examining the existing classification will play an important role in understanding this important class of proteins known to form complexes with DNA.

Firstly, PDB was queried for dsDNA-protein complexes (see Methods) with resolution better than 3 Å. We have built upon the existing groups and families of DNA binding proteins in classification proposed by Luscombe *et al.*, 2000 and selected representatives of each of the families which were also validated using Jack-knifing (leave-one-out) approach. For each of the representatives selected for different families, PSI-BLAST [10] profiles were built using Jump Start PSI-BLAST. The new complexes were individually used as a query against the database of representatives’ profiles using RPS-BLAST [11]. This helped to populate the existing families. The left-out new complexes were clustered and classified based on their biological function and grouped according to the presence of the DNA binding motif in the protein partner. As a result, we were able to classify DNA binding proteins in to 174 families and nine groups.

This newly built two-tier classification where the group indicates the type of DNA binding motif present in the protein partner (except in the Enzyme group where group name indicates that the protein possesses catalytic activity upon binding to DNA) and the family level corresponds to the functional role of the protein, can further be used for performing genome–wide surveys in organism(s) of interest for the presence of DNA-binding proteins.

## Methods

### Selection of DNA-protein complexes from PDB

PDB was searched for DNA-protein complexes having resolution better than 3 Å. The complexes were further filtered for having only double-stranded DNA (dsDNA) and all single-stranded DNA (ssDNA), quadruple DNA (it is higher order structure of nucleic acids which is G-rich and forms four-stranded structure), nucleosomal and previously classified complexes were removed.

### Representatives’ selection for existing 54 families

For all the 54 families from Thornton’s group 2000 classification, representatives were selected and validated. First the pairwise percentage identities were obtained between members of a family using ClustalX [12]. The families having wide percentage identity distribution were carefully analyzed for their representative selection in terms of coverage of each family member as described below. The statistical approach Jack-knifing (leave-one-out approach) was used to validate the selection of representatives in terms of its coverage for being able to pick up all of its own family members. Best representative was selected by providing equal chance to every family member to become the representative and then observing its performance as measured by coverage over its own family (other members of its family are able to pick the representative(s) profile). Either a single member or a combination of members is chosen so as to obtain 100% coverage for a particular family.

(1) $$\begin{array}{l} \text{Coverage of the member i}\\ \text{belonging to family F} \end{array}=\frac{\text{Number of members of family F picking member i profile}}{\text{Total number of members in family F}}$$

In families, where one member was not able to have 100% coverage over its family, more than one member was selected as representatives. For all the selected representatives, PSI-BLAST profiles were built using Jump-start PSI-BLAST at stringent Evalue of 10^-10^, for 20 iterations, where alignment of all family members including the representative was also given as input for profile creation.

Also, the representatives that were selected were tested for their performance by making their PSI-BLAST profiles against dummy database (a database having completely unrelated sequences which are non-DNA binding in nature), so as to ensure that the representative profile is powerful and it is not biased by other sequences, included during profile creation, in its coverage. Therefore, a dummy database helped us to make sure that there were no additional members that might drift the direction of sequence searches. If the selected best representative(s) profile built against this dummy database was still observed to have 100% coverage on its family, it was selected as a true representative.

### Classification of new complexes

The previously existing families were first populated with new complexes with the help of RPS-BLAST, where all profiles of representatives were assembled into a database and the complexes individually were allowed to pick a profile from this database using RPS-BLAST at an E-value of 10^-3^. The single-profile pickers were easily added to the respective family whereas the multiple- or no- profile pickers were dealt with separately.

**Figure 1:**
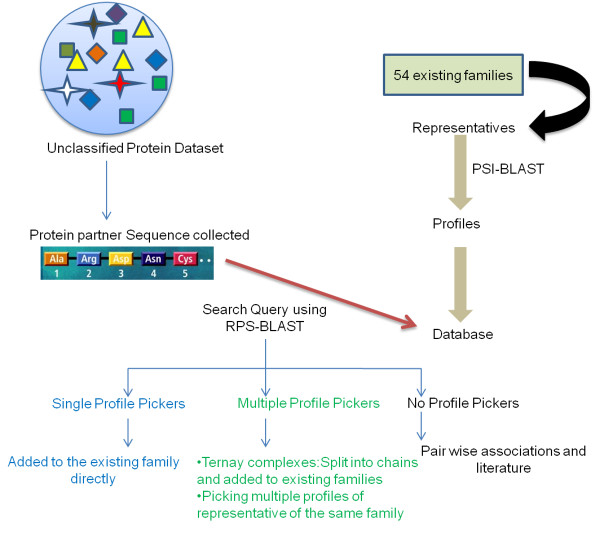
**Methodology.** Schematic of the overall methodology for classifying new complexes using approaches like PSI-BLAST and RPS-BLAST.

Multiple-profile pickers which were observed to be ternary complexes were split into chains and added to the respective families. No-profile pickers were clustered using all-against-all BLAST approach and were added in to new groups and families based on the DNA binding motif and their biological function respectively. Figure 1 depicts the schematic of the overall methodology adopted to classify the new protein-DNA complexes into groups and families.

### New families and their representatives

After the new classification was laid down, for each of the newly formed families and the old families which have undergone expansion in terms of addition of members, new representatives were selected adopting the same approach as mentioned above.

**Figure 2:**
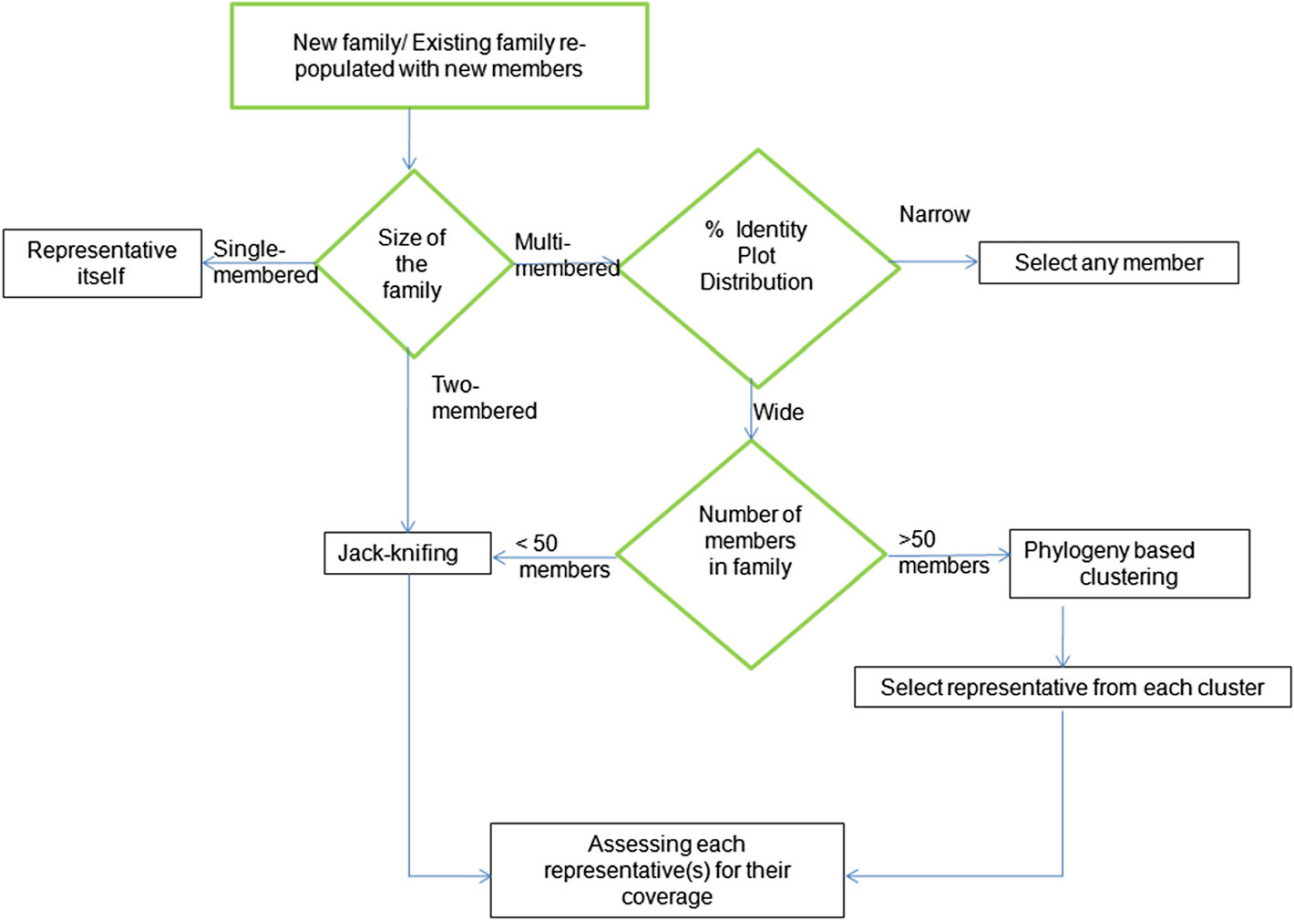
**Representative Selection.** Schematic of the methodology for selecting reprsentatives of new families formed after classification. If the percentage identity plot is narrow, any member of the family is assessed for its coverage, but if the distribution of percent identity is wide, the decision of whether to perform Jack-knifing is dependent on the size of the family.

The best representative of newly formed families was selected using Jack-knifing and phylogeny. The decision to choose either of the one techniques was based on the size of the family and also the distribution of the percent identity plot within the family (Figure 2 depicts the methodology of selecting the representatives). For two-member families, both the members are allowed to behave as a representative and then both are assessed in terms of their coverage for that particular family. If the old representative is able to have 100% coverage on the family, then it was retained as a new representative also.

In the case of multi-member families, the pairwise percentage identity distribution was observed first and in the case of a narrow distribution, any one of the member is assessed for its coverage (as narrow distribution implies the members are nearly identical). Wide percent identity distribution requires decision making for the number of members in a given family. As Jack-knifing is computationally intensive technique [N (N-1) profiles creation for N members], families having <50 members were subjected to Jack-knifing but if the members in family are >50, first clustering was performed and representative “seeds” from cluster(s) were chosen and individually as well as in combination from different clusters, were assessed for their coverage.

## Results and discussion

### Dataset of DNA-protein complex structures

Structures of DNA-protein complexes solved using X-Ray crystallography and resolution better than 3 Å were obtained from PDB. From this dataset, the protein DNA complexes having ssDNA or quadruple DNA was excluded from this classification (see Methods). Some of the complexes were ternary ones having two proteins and DNA molecule; these were split into individual chains and then considered for classification. Thornton and coworkers classified 230 complexes in 2000 and now approximately a four-fold increase in the number of complexes which needs classification was observed (1009 complexes).

As of February 2010, 1354 protein DNA complexes were retrieved from PDB. Already classified complexes (241 (including ternary complexes), [1]), ssDNA, quadruple DNA complexes, ribonucleases, ATP-bound complexes and tri/octa peptides bound to nucleic acid were also removed. This resulted in a dataset of 1009 DNA-protein complexes which need to be classified.

**Figure 3:**
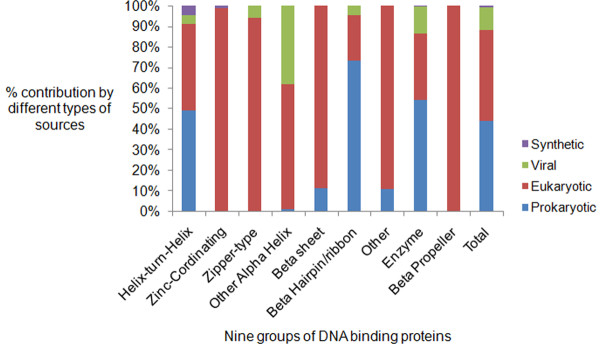
**Source of new nucleic acid-protein complexes.** Source of the classified DNA-protein complexes (including previously classified complexes). Prokaryotic and eukaryotic complexes were almost equal in percentages (44%) and small 11% of the total complexes were viral DNA-protein complexes.

Further, it was observed that there were approximately equal number of prokaryotic and eukaryotic complexes (44% each) but only a very small percent 11% complexes were from viruses (Figure 3).

### Representatives of DNA-binding protein families

For all the existing 54 families in Thornton’s classification [1], best representative was selected with the help of Jack-knifing approach. For these 54 families, 59 representatives were selected ensuring 100% coverage.

**Figure 4:**
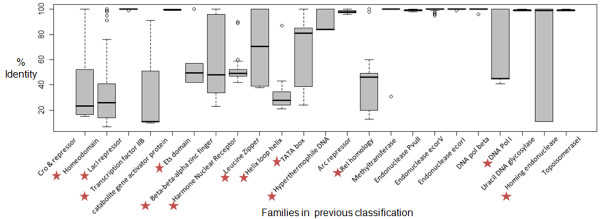
**Percentage Identity distribution for Thornton’s families.** Pairwise percent identity distribution for 23 multi-member Thornton families. (Red stars in front of the family name implies it has wide distribution of percent identity and further the family was subjected to Jack-knifing for selecting the representative).

For 23 out of 54 Thornton’s families, which were multi-membered families (>2 members), pairwise percentage identities were obtained using ClustalX. Figure 4 displays the percent identity distribution for these families in form of Box and whisker plot. For 10 families, a narrow percentage identity range was observed and in these families any one member was assessed for its coverage. In all such cases, one member was observed to have 100% coverage for family. But for families having wide percentage identity range (13 families), each member was given a chance to behave as a representative and later they were assessed for coverage over their family. The total number of representatives selected for 54 families were 59 (Table 1), implying there was more than one representative for some families. These families were Homing endonuclease (2), Homeodomain (3), DNA Polymerase T7 (2) and Transcription factor (2).

**Table 1:** Representatives for previous families 54 existing families (Thornton classification) representatives were selected and were validated using Jack-knifing

**Group**	**Families**	**Representative(s)**
**HTH**		
	Cro & repressor	1LMB
	Homeodomain	1FJL, 1HDD, 6PAX
	LacI repressor	1WET
	Endonuclease Fok1	1FOK
	Gamma Delta resolvase	1GDT
	Hin recombinase	1HCR
	RAP1 family	1IGN
	Prd paired domain	1PDN
	Tc3 transposase	1TC3
	Trp repressor	1TRR
	Diptheria tox repressor	1DDN
	Transcription factor IIB	1D3U
	Interferon regulatory	2IRF
	Catabolite gene activator protein	1RUO
	Transcription factor	1CF7, 3HTS
	Ets domain	1BC8
**Zinc Co-ordinating**		
	β-β-α zinc finger	1ZAA
	Harmone Nuclear Receptor	2NLL
	Loop sheet helix	1TSR
	GAL4 type	1ZME
**Zipper type**		
	Leucine Zipper	1YSA
	Helix loop helix	1AN2
**Other-α Helix**		
	Pappilomavirus 1 E2	2BOP
	Histone	1AOI
	EBNA1 nuclear protein	1B3T
	Skn-1 transcription factor	1SKN
	Cre Recombinase	1CRX
	High Mobility Group	1QRV
	MADS box	1MNM
**β-Sheet**		
	TATA box binding	1YTB
**β-Hairpin/Ribbon**		
	MetJ repressor	1CMA
	Tus replication terminator	1ECR
	Integration host factor	1IHF
	Transcription Factor T-domain	1XBR
	Hyperthermophile DNA	1AZP
	Arc repressor	1PAR
**Other**		
	ReI homology	1SVC
	Stat protein	1BF5
**Enzyme**		
	Methyltransferase	6MHT
	Endonuclease PvuII	3PVI
	Endonuclease ecorV	1RVA
	Endonuclease ecorI	1QPS
	Endonuclease BamHI	3BAM
	Enonuclease V	1VAS
	Dnase I	2DNJ
	DNA mismatch endonuclease	1CW0
	DNA polymerase β	1BPY
	DNA Polymerase I	2BDP
	DNA Polymerase T7	1T7P,1CLQ
	HIV Reverse Transcriptase	2HMI
	Uracil DNA glycosylase	1SSP
	3-Methyladenine DNA glycosylase	1BNK
	Homing endonuclease	1A73, 1BP7
	TopoisomeraseI	1A31

For all the 59 selected representatives, PSI-BLAST profiles were again built against dummy database using the earlier profile creation parameters (as described in Methods). The sequences included in the dummy database were foetal deoxyhemoglobin, relaxin, subtilisin, chymotrypsin and human deoxyhemoglobin. The 59 new profiles built using dummy database were again assessed for their coverage and all were still observed to be best representatives.

### Classification of new complexes

After performing the RPS-BLAST search for each of the 1009 new complexes against database of the family representatives, 444 (~44%) complexes were observed to pick single representative family profile, 118 (~12%) were picking multiple profiles and 447 (~44%) complexes did not pick profile of any of the representatives.

444 new complexes which were able to pick single profiles from database of 59 profiles at Evalue 10^-3^ using RPS-BLAST were added as new members of the existing families and marked as representative associations in the master table of classification [see Additional file 1]. 118 complexes were observed to pick more than one representative’s profile, because of the existence of more than one representative for a family and also 8 of them were ternary complexes (i.e. two proteins bound to DNA [see Additional file 2]. In the first case, when they were picking profiles of the representatives of the same family, the complex was added to that particular family. Ternary complexes were split into different chains and the chains were added to the respective families. There were a total of 12 complexes which were ternary in nature, three were in association with a representative and one was a loner (defined later).

447 complexes, which did not pick any of the representative profile, were checked for all pairwise associations among themselves. 369 of these were observed to form 75 families and rest 78 did not associate with any of the sequences and were termed as loners. For all these 75 families having 369 members, their functional annotations were manually recorded by consulting the literature and the newly formed different families were attributed a status within the existing eight groups and are mentioned as pairwise association [see Additional file 1]; 78 (loners) were likewise mentioned as ‘single-membered families loners’ in master classification chart [see Additional file 1], loners were classified into new families by consulting literature and these references [see Additional file 3] have been marked. The new families were named according to the biological function performed by the respective members. While making these new families in existing groups, one new group ‘β-propeller’ was also realized. Presently, this group has single family which in-turn has two members which are DNA-bound complex of DNA damage repair protein having a seven bladed β-propeller fold.

### New families and their representatives

**Figure 5:**
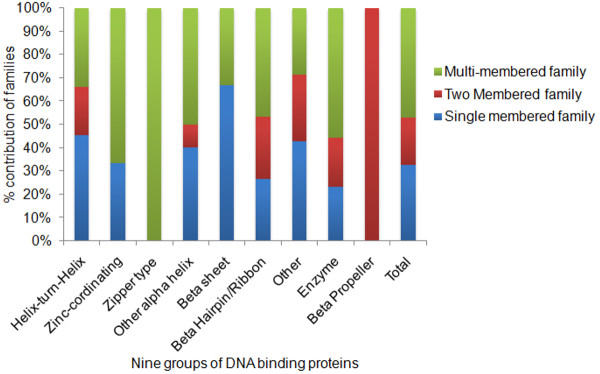
**Percentage distribution of single, two- and multi-membered families in new classification.** Percentage distribution of number of single-, two- and multi-member families in each of the nine groups. Total represents the distribution in all the groups collectively.

The sequence analysis based approach for DNA-protein complexes, as described above, gave rise to classification of DNA-binding proteins into nine groups and 174 families. 59 families (~33%) have only single member, 35 (20%) families have two members and 82 (~47%) families are multi-member families. Figure 5 depicts the percentage distribution of single, two- or multi-member families in each of the nine groups.

The proteins included in the same group exhibit same DNA-binding motif and within the same family they have similar functional roles. The new families were identified by checking the associations of individual proteins with every other protein or with the previously classified protein. The details about the families in nine different groups and PDB codes of the members are recorded in the classification chart [see Additional file 1].

**Figure 6:**
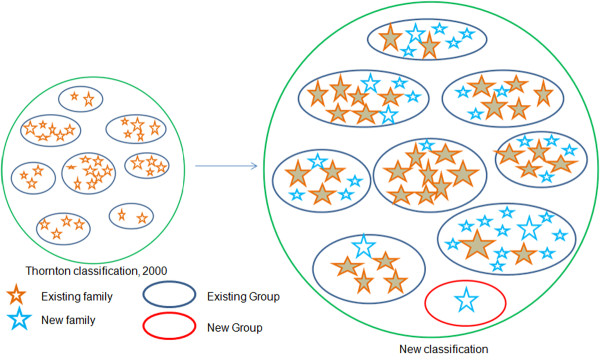
**Schematic of the Classification.** The circles represent the entire pool of DNA-protein complexes. Within the circles, ovals are the different groups of DBP; orange stars are the existing families within groups. These groups vary in their size in terms of number of families present and also the number of members within each family. After our classification, one new group was realized (red oval), also the new members were added to existing families (shaded orange ovals) and also new families were realized as represented by blue stars.

The schematic of the classification represented in Figure 6 highlights the different ways adopted to classify DNA-protein complexes. Table 2 summarizes the modifications performed in Thornton’s families after association of new members. The listed families have been marked up appropriately as renamed or split or merged, as the case may be.

**Table 2:** Modifications in previous families

**Group**	**Existing Families**	
	**Before classification**	**After classification**
**HTH**		
	Cro & repressor	Renamed to Cro and cro like
	Diptheria tox repressor	Renamed to iron dependent repressor
**Zinc Co-ordinating**		
	Harmone Nuclear Receptor	Renamed to Nuclear receptors
	GAL4 type	Renamed to Gal 4 and Gal 4 like
**Zipper type**		
	Leucine Zipper	Has subfamilies bzip1 and bzip2
**Other-α Helix**		
	Cre Recombinase	Renamed to Site specific recombinases
**Other**		
	ReI homology	Renamed to Ig fold like Transcription factor
**Enzyme**		
	DNA polymerase β	Merged and splitted into DNA Polymerase A, B, C, X and Y
	DNA Polymerase I	
	DNA Polymerase T7	
	Uracil DNA glycosylase	Has 3 subfamilies Human UDG, *Xenopus* UDG and *T. thermophilus* UDG

The previously recognised 54 families were split, renamed or merged while classification in order to populate them with the new members. The families listed below have been marked appropriately if they were renamed or split or merged. 17% of the families underwent these treatment. (Rest of the previously recognised families which are not listed did not get change and were populated with new members).

The following are some examples explaining the modifications made to the existing families in order to add the new members:

#### Renaming

While performing classification, six previously recognised families were renamed - Cro and repressor (HTH group), Diphtheria tox repressor (HTH group), Hormone nuclear receptor (Zn co-ordinating group), Gal4 (Zn co-ordinating group), Cre recombinase (Other α-helix group) and ReI homology (Other α-helix group). The names in parentheses indicate their modified names. Table 2 lists these families with their new name incurred upon classification.

Diphtheria tox repressor family was previously having diphtheria tox iron dependent repressor [13], which is known to regulate the toxin coding *tox* gene in *Corynebacterium diphtheriae*. This family was renamed to iron dependent repressors to include iron dependent regulator (IdeR), from *Mycobacterium tuberculosis* which is known to be the functional homolog of diphtheria tox repressor [14].

#### Splitting and merging

Previously existing families, like leucine zipper and Uracil DNA glycosylases, were split into subfamilies. Leucine zipper was observed to have two subfamilies bzip1 and bzip2. In enzymes group, uracil DNA glycosylase family was split into three subfamilies based on the source of the enzyme, human, *Xenopus* or *Thermus thermophilus.*

In the previous classification, DNA polymerases were classified in three families- DNA Pol DNA Pol I and T7 DNA Pol. After new classification, these three families were merged and then split to form five sequence-based families [15], DNA polymerase A, B, C, X and Y.

**Figure 7:**
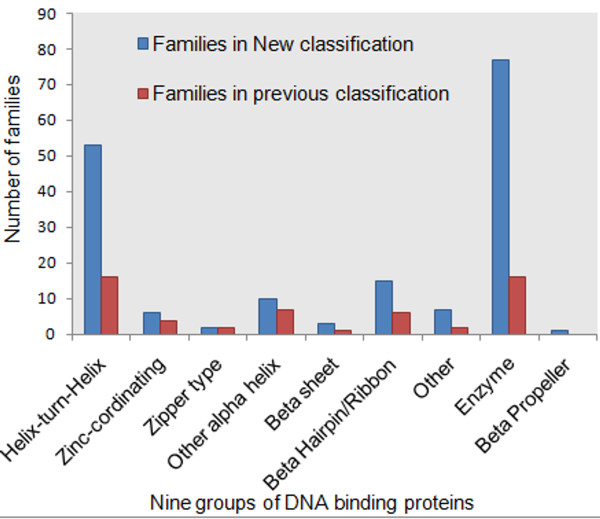
**Distribution of number of families in different groups in both old and new classification.** Total number of families in each group before and after new classification. The highest increase was observed to be the five-fold increase in the total number of families in Enzyme group.

In contrast to the number of existing families in different groups, the maximum fold change in terms of increase in number of families was observed to undergo a five-fold increase in the Enzyme group. However, in groups HTH, β-sheet and ‘Other’, an approximately three-fold increase in the number of families was observed. Three groups, Zinc coordinating, Zipper type and Other α-helix, were not observed to experience significant increase in the number of families during the re-classification in comparison to the number of previously existing families. Figure 7 shows the total number of families within each group before and after our classification.

**Figure 8:**
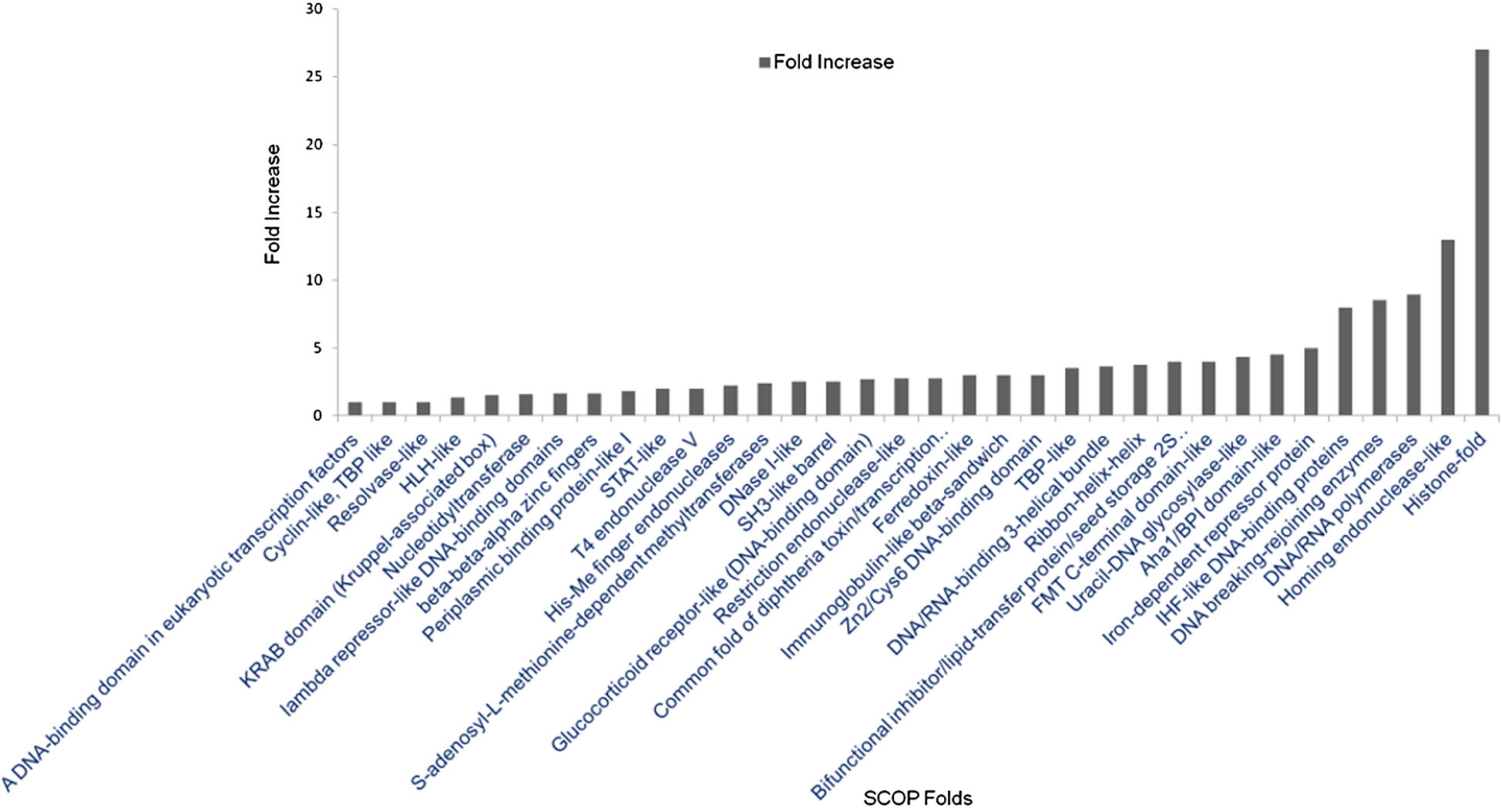
**Common SCOP folds in old and new classification34 common folds in both old and new classification complexes.** Number of members possessing these folds expanded in new classification compared to old classification. The fold increase in the number of members with each of these 34 folds is plotted. Maximum fold increase of 27 was observed in Histone family

The new families were also examined for their folds as ascribed to them by SCOP 1.75 [16], and the folds were recorded [see Additional file 1. Although SCOP is a highly updated database, we realised that ~30% of the entries (PDB IDs) were not included in SCOP 1.75 due to newer PDB entries. 34 SCOP folds were common to both new and old classification and they experienced an expansion in the number of complexes. The fold change in these 34 common folds is represented in Figure 8. The number of members, belonging to both old and new classification possessing each of the common 34 folds is summarised [see Additional file 4. The top three folds, experiencing maximum expansion in terms of members possessing them, were Histone, Homing endonuclease and DNA/RNA Polymerase - truly reflecting the maximum increase in the number of members and families in enzymes group. Therefore, expansion in the existing families was seen to a maximum extent in the families of enzyme group which have property to bind to DNA and then carry out an enzymatic activity.

**Figure 9:**
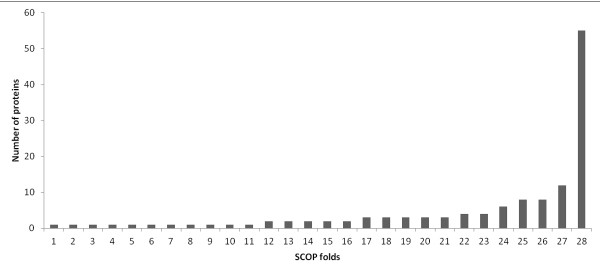
**SCOP folds only in new classification28 folds present only in newly classified complexes.** The fold exhibited by maximum number of newly classified complexes are those which are involved in DNA damage repair functions like Lesion bypass DNA Polymerase, MutS domain, Glycosylase. Numbers represent the respective names of SCOP fold in the Figure 1. ATP-dependent DNA ligase DNA-binding domain, 2. Cryptochrome/photolyase FAD-binding domain, 3. DNA-clamp, 4. Double-stranded β-helix, 5. GCM domain, 6. Hcp1-like, 7. Metallo-dependent phosphatases, 8. Phage replication organizer domain, 9. SPOC domain-like, vWA-like, 10. Thioredoxin fold, 11. Type II DNA topoisomerase ,12. DNA-binding domain of intron-encoded endonucleases, 13. Phospholipase D/nuclease, 14. Replication modulator SeqA, C-terminal DNA-binding domain, 15. SMAD MH1 domain, 16. UDP-Glycosyltransferase/glycogen phosphorylase, 17. N-terminal domain of MutM-like DNA repair proteins, 18. P-loop containing nucleoside triphosphate hydrolases, 19. SAM domain-like, 20. SRF-like, 21. Zinc finger design, 22. Origin of replication-binding domain, RBD-like, 23. Ribonuclease H-like motif, 24. PUA domain-like, 25. DNA-glycosylase, 26. Putative DNA-binding domain, 27. DNA-repair protein MutS, domain III, 28. Lesion bypass DNA polymerase (Y-family)

Also, there were 28 folds which were present only in new complexes, suggesting emergence of structures of complexes performing new functions (Figure 9). The proteins possessing DNA-repair function is present exclusively in the newly classified complexes like Y-family DNA polymerases which are known to bypass a lesion in DNA, DNA glycosylases and MutS DNA-repair proteins.

**Figure 10:**
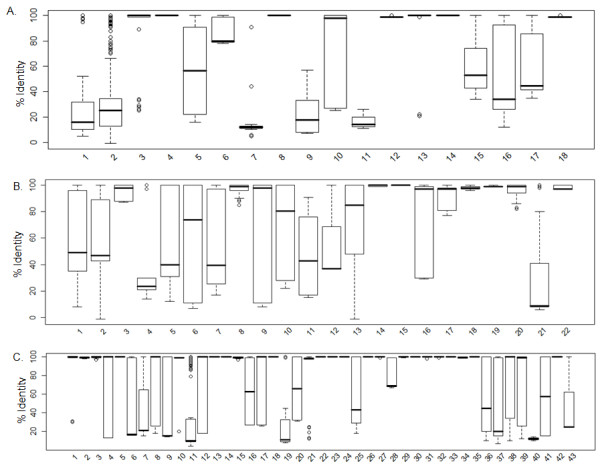
**Pairwise percentage identity distribution for new families.** (**A**). Boxplot for pairwise percent distribution for 18 multi-member families of group HTH, the following are the family names 1. *Cro and Cro like 2. *Homeodomain 3. LacI repressor 4. Hin recombinase 5. *RAP1 6. *Iron Dependent repressor 7. *Transcription factor IIB/IIA 8. NarL transcription factor 9. *Tn5 Transposase 10. *MutS 11. *Tetracycline Repressor 12. Interferon regulatory factor 13. *Catabolite gene activator protein 14. Transcription factor 15. *Ets domain 16. *Z-α domain 17. *Forkhead TF 18. Transcription activator BMRR (**B**). Boxplot for pairwise percent distribution for 20* multi-member families of group Zinc co-ordinating, Zipper type, Other α-helix, β-sheet, β-hairpin/ribbon and Other. The following are the family names- 1. *Zinc-coordinating-β-β-α-zinc finger 2. *Zinc-coordinating-Nuclear Receptors 3. *Zinc-coordinating-Loop-sheet-helix 4. *Zinc-coordinating-gal4 5. *Zipper-type-bzip1 6. *Zipper-type-bzip2 7. *Zipper-type-Helix-loop-helix 8. 8Other α-helix -histone 9. *Other α-helix -Site specific recombinases 10. *Other α-helix -High mobility group 11. Other α-helix -MADS box 12. *Other α-helix -CUT domain 13. *β-sheet-TATA box-binding 14. *β-hairpin-MetJ repressor 15. β-hairpin-Tus replication terminator 16. *β-hairpin-Integration host factor 17. *β-hairpin-Hyperthermophile DNA-BP 18. *β –hairpin-Arc repressor 19. β-hairpin-Omega Repressor 20. *β-hairpin-SRA Domain 21. *Other-Ig fold like TF 22. *Other-Seq A*Total number of plots are 21 in Figure 9(B) as bzip1 and bzip2 boxplots are different but they are subfamilies of single Leucine zipper family (**C**). Boxplot for pairwise percent distribution for 43 multi-member families of group enzymes. The following are the family names- 1. Methyltransferase 2. Endonuclease PvuII 3. Endonuclease ecorV 4. *Endonuclease ecorI 5. Endonuclease BamHI 6. *Enonuclease V 7. *Dnase I 8. *HIV reverse transcriptase 9. *Uracil-DNA glycosylase 10. 3-Methyladenine DNA glycosylase 11. *Homing endonuclease 12. *Topoisomerase I 13. T7 RNA Pol 14. N4 RNA Pol 15. HincII restriction endonuclease 16. *Endonuclease III and MutY 17. *DNA Photolyase 18. α-glucosyl transferase 19. *Helicase 20. *Thymine DNA-glycosylase 21. 8-oxoguanine DNA glycosylase 22. ALKA 23. Phi 6 RNA Pol 24. β-Glucosyltransferase 25. *Endonuclease VIII and MutM 26. Human tyrosyl-DNA phosphodiesterase 27. Relaxase TrwC 28. *Nuclease-Colicin 29. Endonuclease IV 30. Excisionase (Xis) 31. ISHp608 Transposase 32. AlkB 33. Restriction Endonuclease HinP1I 34. ABH2 35. Restriction endonuclease SgrAI 36. *Family A Polymerases 37. *Family B Polymerases 38. *Family X Polymerases 39. *Family Y Polymerases 40. *DNA Ligase 41. *Family C polymerases 42. Mtaq 1 methylase 43.* DAM(S*tars in front of the family name implies it has wide distribution of percent identity and further the family was subjected to Jack-knifing for selecting the representative)*

It was observed that for all the groups, in total, there were 57 single-member, 35 two- member and 82 multi-member families. New representatives were also selected for these 174 new families. For 57 single-member families, the member itself was a representative. In two-member families, equal chance to each member was given to become a representative and the one having 100% coverage was selected as representative. For multi-member families, the pairwise percentage identity distribution in the form of box-plot is represented in Figure 10. Out of 82 multi-member families, 32 were observed as having narrow percent identity distribution, whereas 50 families (marked with star Figure 10) were having wide distribution of percentage identity. For 47 families, leave-one-out approach was adopted to find best representative. There were three families (out of 50) with wide percentage identities with >50 members- Family A DNA Pol, Family X DNA Pol and Family Y DNA Pol, where clustering was performed followed by assessing the representative from every cluster both individually and in combination to assess its coverage.

### Old vs. new representatives

While selecting the new representatives, care was taken to retain the previously chosen representative, if it showed 100% coverage for the family including the newly added members. As a result, it was observed that 75% (for 38 families out of 51) of the previously chosen representatives were retained as family representatives even after adding the new members [see Additional file 5]. In total, 191 representatives were identified for 174 families [see Additional file 6].

## Conclusions

Protein nucleic acid complexes form the most vital interacting macromolecular pairs existing in the biological cell. It governs number of cellular processes and hence helps in maintaining the normal physiological state of the cell.

Here, we have investigated the existing DNA-protein complexes in the PDB (Feb2010) and provided a systematic two-tier protein-centric classification for them. To achieve this, we have looked upon and studied the existing classification [1]. But due to nearly exponentially increasing growth of PDB [17], there is a need to revisit the existing classification.

The main features of the classification we propose are:

1. The number of complexes classified is ~5 times (1009 vs. 230) more than the number of DNA-protein complexes classified previously. There were approximately equal number of complexes from prokaryotic as well as eukaryotic sources, but only little above 11% of the complexes were having viral proteins.

2. It is a two-tier classification at group and family level. At the first level, group defines the DNA-binding motif present except in the Enzyme group, where any protein with the capability to bind to DNA and exhibiting enzymatic activity was placed. At the family level, proteins were grouped on the basis of their biological function by checking associations of individual proteins with each other or with the previously classified protein.

3. A new group ‘β-Propeller’ is brought in, presently having only one family- DDB1-DDB2, which plays a role in UV DNA damage recognition using its seven bladed β-propeller [18].

4. The number of families has increased to ~3 times (174 vs. 54) by virtue of the increase in the number of DNA-protein complexes deposited in PDB (a 60% increase with respect to increasing entries of DNA-bound protein complexes in PDB). There was a five-fold increase in the number of families in Enzyme group alone and this was accompanied by a large increase in the number of complexes (number of complexes in Enzymes group increased to 714 from Thornton’s 113) in Enzyme group after our classification. ~67% families have more than one member in the new classification. This indicates some groups are growing fast in terms of the family numbers faster than the others, which can be explained due to several reasons. Firstly, this can be due to the higher utilisation of some DNA-binding motifs over the other: for example, helix-turn-helix motif is most frequently represented motif. However, the group of Enzymes has more families due to the diverse nature of biological function performed by the proteins which possess catalytic activity upon binding DNA. Secondly, there are some specific motifs like Zipper type which are meant to perform not-so-diverse functions so the numbers of families in such groups tend to be less. There is also an inherent bias or preference for certain structure targets that affects the number of families in the group. For example, presently in the field more emphasis is on DNA-repair proteins, proteins with implications in diseases etc.

While performing classification, 17% of the existing families were observed to have undergone either splitting or renaming in order to make add more complexes to the family (Percentage marked, Table 2). The analysis of folds present in complexes of old and new classification reflected that maximum fold increase was in Histone and then in folds which are present in enzymes like Homing endonuclease-like, DNA/RNA Polymerase and DNA-breaking and rejoining.

There were also new folds observed which were present only in new complexes. This is suggestive of the growth of PDB over a year of 10 years, both in terms of number of complexes and the folds present in the structures which are getting deposited. The folds which were noted to appear only in the new classification were ones known to perform function of DNA damage repair like DNA glycosylase, Lesion bypass DNA Polymerase (Y-family) and Mut S domain.

The classification of DNA-binding proteins will provide a very useful insight in exploring further the sequence-to-structure-to-function paradigm, also about the interaction between protein and its respective DNA partner to govern and fine-tune the effecter function of the cell. The current classification will help to understand the given complex of interest in terms of to which group (DNA-binding motif) and family (biological function) it belongs to.

Unlike the structure-based classification by Thornton and coworkers, that formed a strong platform for the current study, we have now adopted a pure sequence-based classification strategy owing to the large number of structural entries added on. Also, due to strong structural convergence and fine-tuned sequence changes in and near the ligand-binding site, simple structural comparisons may be insufficient in some cases. To compare our approach with the structural alignment methods, we are highlighting an example where it is difficult to decide the cut-off RMSD value for a particular family [see Additional files 7, 8 and 9], wherein all neighbouring families which are reported to have same SCOP fold i.e. DNA/RNA binding three-helical bundle.

We performed a case study on Homeodomain family belonging to Helix-turn-helix group. All pairwise structural alignments for 34 members in Homeodomain family was performed using rigid-body superposition and the pairwise RMSD values are depicted in Additional file 7 (7 entries out of 34 were heterodimers and for them chains were split and then the structural alignment was performed, resulting in overall 41 chains and 820 pairwise alignments). For PDB ID 1JGG (marked in green, [see Additional file 7]), RMSD with five of its own classified family members was observed to be >2 Å. The RMSD value of 2 Å was also observed for 1JGG, in a non-specific manner, with representative for another family Trp Repressor (HTH group, same SCOP as Homeodomain), 1TRR. Also, RMSD of 2.1 Å was observed between 1JGG and 1TC3 (Representative for family TC3 transposase family, HTH group and have same SCOP fold as Homeodomain) (Additional file 9). This exemplifies that RMSD values as a result of structural alignment can pose a difficulty in deciding a cut-off value for a particular family and may not be a useful single determinant for association of new entries to previously existing protein structural entries. On the other hand, if we compare it with our profile-based approach using RPS-BLAST, 1JGG was observed to associate specifically only to the profile of Homeodomain proteins namely 1FJL (at E-value 3e^-8^) and 1HDD (at E-value 6e^-13^) [see Additional file 8]. By observing the structural alignment and RMSD values alone for above mentioned pairs, it becomes difficult to identify a particular family member (all RMSD values > =2.5 Å (47 pairwise RMSD) are marked in red [see Additional file 7]).

Next, we applied RPS-BLAST to associate large number of gene products with our database of sequences of proteins that bind to DNA. Where simple approaches like PSI-BLAST was not able to identify associations to DNA-protein families, RPS-BLAST and HMM methods provide unique associations when run on the whole genome of *Arabidopsis thaliana* [see Additional file 10]. We also hope that such searches can be extended to sequence-centric databases like genomes of model organisms in the future.

In future, this classification can aid in performing several genome-wide studies which can be performed in various genomes of interest to study the expansion or disappearance of a particular family in specific lineage. This will provide an insight into various modes of regulation existing in different lineages at the level of proteins known to interact with DNA. Also, utilizing the various features of all DNA-binding proteins, SVM-based machine learning algorithms can be developed to predict whether a sequence of interest exhibits DNA-binding property or not. We can make even more specific predictions, such as given protein sequence belongs to which particular group and family can be identified by extracting the family specific features. Also, classification can be extended to include sequence families of DNA binding proteins which will aid in complete understanding of the features of this class of proteins. It will also be worthwhile to build classification schemes for other proteins which are involved in governing cellular integrity and its function.

## Competing interests

The author declares that they have no competing interests.

## Authors’ contributions

RS designed the project and conceived the experiments. SM carried out coding and scripting and performed the entire analysis. SM drafted the manuscript and RS provided critical comments to improve it. All authors read and approved the final manuscript.

## Supplementary Material

Additional file 1 **Master table of the classification.** Association of additional and new members to pre-existing families. Click here for file

Additional file 2 **List of references that describe ‘loners’ protein-DNA complex [**[19-92]**].** Click here for file

Additional file 3 Ternary protein-DNA complexes. Click here for file

Additional file 4 The number of complexes in both old and new classification possessing each of the common 34 folds. Click here for file

Additional file 5 **New vs. Old representatives.** 38/51* cases where old representative was still observed to have 100%coverage on the family even after addition of new members. *The number of old families here is 54 but here it is taken as 51 as the three polymerase families (T7 DNA Pol, DNA Pol β and DNA Pol I) were split in the new classification. Click here for file

Additional file 6 **Representatives for new families.** New families with their selected representatives (validated using Jack-knifing). Click here for file

Additional file 7 Pairwise RMSD values obtained using MATT for all members of Homeodomain family belonging to HTH group. Click here for file

Additional file 8 RPS-BLAST alignment of new member of Homeodomain family (1JGG) with Homeodomain representatives (1HDD and 1FJL). Click here for file

Additional file 9 **Structural superposition using MATT.** Structural alignment for 1JGG (Homeodomain new member) with 1TRR (Trp Repressor representative), 1TC3 (Tc3 transposase representative) and 6PAX (Homeodomain representative)]. Click here for file

Additional file 10 **Unique hits obtained by profile-based methods.** Some examples of DNA-binding proteins identified using only profile-based searches are observed during genome-wide survey in *Arabidopsis thaliana*. Click here for file
