# Supplementary material for: Re-visiting protein-centric two-tier classification of existing DNA-protein complexes
Source: BMC Bioinformatics. 2012 Jul 16;13:165. doi: 10.1186/1471-2105-13-165 (PMC3472317; doi:10.1186/1471-2105-13-165)
Supplement: Additional file 2 — List of references that describe ‘loners’ protein-DNA complex [[19-92]]. [file 1471-2105-13-165-S2.pdf]

List of references that describe ‘loners’ DNA-protein complex:

1. Iyaguchi D, Yao M, Watanabe N, Nishihira J, Tanaka I: **DNA Recognition Mechanism of the ONECUT Homeodomain of Transcription Factor HNF-6**. *Structure* 2007, **15**:75-8310.1016/j.str.2006.11.004.

2. Chi Y, Frantz JD, Oh B, Hansen L, Dhe-Paganon S, Shoelson SE: **Diabetes mutations delineate an atypical POU domain in HNF-1alpha**. *Mol. Cell* 2002, **10**:1129-1137.

3. Yousef MS, Matthews BW: **Structural Basis of Prospero-DNA InteractionImplications for Transcription Regulationin Developing Cells**. *Structure* 2005, **13**:601-60710.1016/j.str.2005.01.023.

4. Tawaramoto MS, Park S, Tanaka Y, Nureki O, Kurumizaka H, Yokoyama S: **Crystal structure of the human centromere protein B (CENP-B) dimerization domain at 1.65-A resolution**. *J. Biol. Chem* 2003, **278**:51454-5146110.1074/jbc.M310388200.

5. Orth P, Schnappinger D, Hillen W, Saenger W, Hinrichs W: **Structural basis of gene regulation by the tetracycline inducible Tet repressor-operator system**. *Nat. Struct. Biol* 2000, **7**:215-21910.1038/73324.

6. Schumacher MA, Miller MC, Grkovic S, Brown MH, Skurray RA, Brennan RG: **Structural basis for cooperative DNA binding by two dimers of the multidrug-binding protein QacR**. *EMBO J* 2002, **21**:1210-121810.1093/emboj/21.5.1210.

7. Itou H, Watanabe N, Yao M, Shirakihara Y, Tanaka I: **Crystal Structures of the Multidrug Binding Repressor Corynebacterium glutamicum CgmR in Complex with Inducers and with an Operator**. *Journal of Molecular Biology* 2010, **403**:174-18410.1016/j.jmb.2010.07.042.

8. Komori H, Matsunaga F, Higuchi Y, Ishiai M, Wada C, Miki K: **Crystal structure of a prokaryotic replication initiator protein bound to DNA at 2.6 A resolution**. *EMBO J* 1999, **18**:4597-460710.1093/emboj/18.17.4597.

9. Schumacher MA, Funnell BE: **Structures of ParB bound to DNA reveal mechanism of partition complex formation**. *Nature* 2005, **438**:516-51910.1038/nature04149.

10. Williams CE, Grotewold E: **Differences between plant and animal Myb domains are fundamental for DNA binding activity, and chimeric Myb domains have novel DNA binding specificities**. *J. Biol. Chem* 1997, **272**:563-571.

11. König B, Müller JJ, Lanka E, Heinemann U: **Crystal structure of KorA bound to operator DNA: insight into repressor cooperation in RP4 gene regulation**. *Nucleic Acids Res* 2009, **37**:1915-192410.1093/nar/gkp044.

12. Shen A, Higgins DE, Panne D: **Recognition of AT-Rich DNA Binding Sites by the MogR Repressor**. *Structure* 2009, **17**:769-77710.1016/j.str.2009.02.018.

13. Lee KS, Bumbaca D, Kosman J, Setlow P, Jedrzejas MJ: **Structure of a protein–DNA complex essential for DNA protection in spores of Bacillus species**. *Proceedings of the*

National Academy of Sciences 2008, **105**:2806.

14. Lane WJ, Darst SA: **The structural basis for promoter -35 element recognition by the group IV sigma factors**. *PLoS Biol* 2006, **4**:e26910.1371/journal.pbio.0040269.

15. Fuhrmann J, Schmidt A, Spiess S, Lehner A, Turgay K, Mechtler K, Charpentier E, Clausen T: **McsB Is a Protein Arginine Kinase That Phosphorylates and Inhibits the Heat-Shock Regulator CtsR**. *Science* 2009, **324**:1323-132710.1126/science.1170088.

16. McGeehan JE, Streeter SD, Thresh SJ, Ball N, Ravelli RB, Kneale GG: **Structural analysis of the genetic switch that regulates the expression of restriction-modification genes**. *Nucleic acids research* 2008, **36**:4778.

17. Fujikawa N, Kurumizaka H, Nureki O, Terada T, Shirouzu M, Katayama T, Yokoyama S: **Structural basis of replication origin recognition by the DnaA protein**. *Nucleic Acids Res* 2003, **31**:2077-2086.

18. Zhao H, Msadek T, Zapf J, Madhusudan, Hoch JA, Varughese KI: **DNA complexed structure of the key transcription factor initiating development in sporulating bacteria**. *Structure* 2002, **10**:1041-1050.

19. Khare D, Ziegelin G, Lanka E, Heinemann U: **Sequence-specific DNA binding determined by contacts outside the helix-turn-helix motif of the ParB homolog KorB**. *Nat. Struct. Mol. Biol* 2004, **11**:656-66310.1038/nsmb773.

20. He C, Hus J, Sun LJ, Zhou P, Norman DPG, Dötsch V, Wei H, Gross JD, Lane WS, Wagner G, Verdine GL: **A methylation-dependent electrostatic switch controls DNA repair and transcriptional activation by E. coli ada**. *Mol. Cell* 2005, **20**:117-12910.1016/j.molcel.2005.08.013.

21. Ha SC, Kim D, Hwang HY, Rich A, Kim YG, Kim KK: **The crystal structure of the second Z-DNA binding domain of human DAI (ZBP1) in complex with Z-DNA reveals an unusual binding mode to Z-DNA**. *Proceedings of the National Academy of Sciences* 2008, **105**:20671.

22. Ha SC, Lokanath NK, Van Quyen D, Wu CA, Lowenhaupt K, Rich A, Kim Y, Kim KK: **A poxvirus protein forms a complex with left-handed Z-DNA: crystal structure of a Yatapoxvirus Zalpha bound to DNA**. *Proc. Natl. Acad. Sci. U.S.A* 2004, **101**:14367-1437210.1073/pnas.0405586101.

23. Schumacher MA, Lau AOT, Johnson PJ: **Structural basis of core promoter recognition in a primitive eukaryote**. *Cell* 2003, **115**:413-424.

24. Yokoyama K, Ishijima SA, Koike H, Kurihara C, Shimowasa A, Kabasawa M, Kawashima T, Suzuki M: **Feast/Famine Regulation by Transcription Factor FL11 for the Survival of the Hyperthermophilic Archaeon Pyrococcus OT3**. *Structure* 2007, **15**:1542-155410.1016/j.str.2007.10.015.

25. Huang N, De Ingeniis J, Galeazzi L, Mancini C, Korostelev YD, Rakhmaninova AB, Gelfand MS, Rodionov DA, Raffaelli N, Zhang H: **Structure and Function of an ADP-**

Ribose-Dependent Transcriptional Regulator of NAD Metabolism. *Structure* 2009, **17**:939-95110.1016/j.str.2009.05.012.

26. Cherney LT, Cherney MM, Garen CR, Lu GJ, James MN: **Crystal structure of the arginine repressor protein in complex with the DNA operator from Mycobacterium tuberculosis**. *Journal of molecular biology* 2008, **384**:1330–1340.

27. Garnett JA, Marincs F, Baumberg S, Stockley PG, Phillips SEV: **Structure and function of the arginine repressor-operator complex from Bacillus subtilis**. *J. Mol. Biol* 2008, **379**:284-29810.1016/j.jmb.2008.03.007.

28. Gajiwala KS, Chen H, Cornille F, Roques BP, Reith W, Mach B, Burley SK: **Structure of the winged-helix protein hRFX1 reveals a new mode of DNA binding**. *Nature* 2000, **403**:916-92110.1038/35002634.

29. Blanco AG, Sola M, Gomis-Rüth FX, Coll M: **Tandem DNA recognition by PhoB, a two-component signal transduction transcriptional activator**. *Structure* 2002, **10**:701-713.

30. Watanabe S, Kita A, Kobayashi K, Miki K: **Crystal structure of the [2Fe-2S] oxidative-stress sensor SoxR bound to DNA**. *Proc. Natl. Acad. Sci. U.S.A* 2008, **105**:4121-412610.1073/pnas.0709188105.

31. Schumacher MA, Hurlburt BK, Brennan RG: **Crystal structures of SarA, a pleiotropic regulator of virulence genes in S. aureus**. *Nature* 2001, **409**:215–219.

32. Sabogal A, Lyubimov AY, Corn JE, Berger JM, Rio DC: **THAP proteins target specific DNA sites through bipartite recognition of adjacent major and minor grooves**. *Nat Struct Mol Biol* 2009, **17**:117-12310.1038/nsmb.1742.

33. Bates DL, Chen Y, Kim G, Guo L, Chen L: **Crystal structures of multiple GATA zinc fingers bound to DNA reveal new insights into DNA recognition and self-association by GATA**. *J. Mol. Biol* 2008, **381**:1292-130610.1016/j.jmb.2008.06.072.

34. Cohen SX, Moulin M, Hashemolhosseini S, Kilian K, Wegner M, M\üller CW: **Structure of the GCM domain–DNA complex: a DNA-binding domain with a novel fold and mode of target site recognition**. *The EMBO Journal* 2003, **22**:1835–1845.

35. Schumacher MA: **The Structure of a CREB bZIPmiddle dotSomatostatin CRE Complex Reveals the Basis for Selective Dimerization and Divalent Cation-enhanced DNA Binding**. *Journal of Biological Chemistry* 2000, **275**:35242-3524710.1074/jbc.M007293200.

36. Fujii Y, Shimizu T, Toda T, Yanagida M, Hakoshima T: **Structural basis for the diversity of DNA recognition by bZIP transcription factors**. *Nature Structural & Molecular Biology* 2000, **7**:889–893.

37. Kurokawa H, Motohashi H, Sueno S, Kimura M, Takagawa H, Kanno Y, Yamamoto M, Tanaka T: **Structural Basis of Alternative DNA Recognition by Maf Transcription Factors**. *Molecular and Cellular Biology* 2009, **29**:6232-624410.1128/MCB.00708-09.

38. Longo A, Guanga GP, Rose RB: **Crystal Structure of E47−NeuroD1/Beta2 bHLH Domain−DNA Complex: Heterodimer Selectivity and DNA Recognition**. *Biochemistry* 2008, **47**:218-22910.1021/bi701527r.

39. Bradley CM, Ronning DR, Ghirlando R, Craigie R, Dyda F: **Structural basis for DNA bridging by barrier-to-autointegration factor**. *Nat Struct Mol Biol* 2005, **12**:935-93610.1038/nsmb989.

40. Albert A, Muñoz-Espín D, Jiménez M, Asensio JL, Hermoso JA, Salas M, Meijer WJJ: **Structural basis for membrane anchorage of viral phi29 DNA during replication**. *J. Biol. Chem* 2005, **280**:42486-4248810.1074/jbc.C500429200.

41. Lindner SE, De Silva EK, Keck JL, Llinás M: **Structural determinants of DNA binding by a P. falciparum ApiAP2 transcriptional regulator**. *Journal of molecular biology* 2010, **395**:558–567.

42. Sidote DJ, Barbieri CM, Wu T, Stock AM: **Structure of the Staphylococcus aureus AgrA LytTR Domain Bound to DNA Reveals a Beta Fold with an Unusual Mode of Binding**. *Structure* 2008, **16**:727-73510.1016/j.str.2008.02.011.

43. Schumacher MA, Glover TC, Brzoska AJ, Jensen SO, Dunham TD, Skurray RA, Firth N: **Segrosome structure revealed by a complex of ParR with centromere DNA**. *Nature* 2007, **450**:1268-127110.1038/nature06392.

44. Zhou Y, Larson JD, Bottoms CA, Arturo EC, Henzl MT, Jenkins JL, Nix JC, Becker DF, Tanner JJ: **Structural basis of the transcriptional regulation of the proline utilization regulon by multifunctional PutA**. *J. Mol. Biol* 2008, **381**:174-18810.1016/j.jmb.2008.05.084.

45. Min J, Pavletich NP: **Recognition of DNA damage by the Rad4 nucleotide excision repair protein**. *Nature* 2007, **449**:570-57510.1038/nature06155.

46. Walker JR, Corpina RA, Goldberg J: **Structure of the Ku heterodimer bound to DNA and its implications for double-strand break repair**. *Nature* 2001, **412**:607-61410.1038/35088000.

47. Ho KL, McNae IW, Schmiedeberg L, Klose RJ, Bird AP, Walkinshaw MD: **MeCP2 Binding to DNA Depends upon Hydration at Methyl-CpG**. *Molecular Cell* 2008, **29**:525-53110.1016/j.molcel.2007.12.028.

48. Badia D, Camacho A, Pérez-Lago L, Escandon C, Salas M, Coll M: **The Structure of Phage 29 Transcription Regulator p4-DNA Complex Reveals an N-Hook Motif for DNA Binding**. *Molecular cell* 2006, **22**:73–81.

49. Metz AH, Hollis T, Eichman BF: **DNA damage recognition and repair by 3-methyladenine DNA glycosylase I (TAG)**. *EMBO J* 2007, **26**:2411-242010.1038/sj.emboj.7601649.

50. Spiegel PC, Chevalier B, Sussman D, Turmel M, Lemieux C, Stoddard BL: **The**

structure of I-CeuI homing endonuclease: Evolving asymmetric DNA recognition from a symmetric protein scaffold. *Structure* 2006, **14**:869-88010.1016/j.str.2006.03.009.

51. Shen BW, Landthaler M, Shub DA, Stoddard BL: **DNA binding and cleavage by the HNH homing endonuclease I-HmuI**. *J. Mol. Biol* 2004, **342**:43-5610.1016/j.jmb.2004.07.032.

52. Frei C, Gasser SM: **RecQ-like helicases: the DNA replication checkpoint connection**. *J. Cell. Sci* 2000, **113 (Pt 15)**:2641-2646.

53. Faucher F, Wallace SS, Doublié S: **The C-terminal lysine of Ogg2 DNA glycosylases is a major molecular determinant for guanine/8-oxoguanine distinction**. *J. Mol. Biol* 2010, **397**:46-5610.1016/j.jmb.2010.01.024.

54. Hashimoto H, Shimizu T, Imasaki T, Kato M, Shichijo N, Kita K, Sato M: **Crystal structures of type II restriction endonuclease EcoO109I and its complex with cognate DNA**. *J. Biol. Chem* 2005, **280**:5605-561010.1074/jbc.M411684200.

55. Newman M, Murray-Rust J, Lally J, Rudolf J, Fadden A, Knowles PP, White MF, McDonald NQ: **Structure of an XPF endonuclease with and without DNA suggests a model for substrate recognition**. *EMBO J* 2005, **24**:895-90510.1038/sj.emboj.7600581.

56. Biertümpfel C, Yang W, Suck D: **Crystal structure of T4 endonuclease VII resolving a Holliday junction**. *Nature* 2007, **449**:616-62010.1038/nature06152.

57. Sukackaite R, Grazulis S, Bochtler M, Siksnys V: **The recognition domain of the BpuJI restriction endonuclease in complex with cognate DNA at 1.3-A resolution**. *J. Mol. Biol* 2008, **378**:1084-109310.1016/j.jmb.2008.03.041.

58. Deibert M, Grazulis S, Janulaitis A, Siksnys V, Huber R: **Crystal structure of MunI restriction endonuclease in complex with cognate DNA at 1.7 A resolution**. *EMBO J* 1999, **18**:5805-581610.1093/emboj/18.21.5805.

59. van der Woerd MJ, Pelletier JJ, Xu S, Friedman AM: **Restriction enzyme BsoBI-DNA complex: a tunnel for recognition of degenerate DNA sequences and potential histidine catalysis**. *Structure* 2001, **9**:133-144.

60. Newman M, Lunnen K, Wilson G, Greci J, Schildkraut I, Phillips SE: **Crystal structure of restriction endonuclease BglI bound to its interrupted DNA recognition sequence**. *EMBO J* 1998, **17**:5466-547610.1093/emboj/17.18.5466.

61. Deibert M, Grazulis S, Sasnauskas G, Siksnys V, Huber R: **Structure of the tetrameric restriction endonuclease NgoMIV in complex with cleaved DNA**. *Nat. Struct. Biol* 2000, **7**:792-79910.1038/79032.

62. Huai Q, Colandene JD, Topal MD, Ke H: **Structure of NaeI-DNA complex reveals dual-mode DNA recognition and complete dimer rearrangement**. *Nat. Struct. Biol* 2001, **8**:665-66910.1038/90366.

63. Campbell EA, Muzzin O, Chlenov M, Sun JL, Olson CA, Weinman O, Trester-Zedlitz

ML, Darst SA: **Structure of the bacterial RNA polymerase promoter specificity sigma subunit**. *Mol. Cell* 2002, **9**:527-539.

64. Hickman AB, Ronning DR, Perez ZN, Kotin RM, Dyda F: **The nuclease domain of adeno-associated virus rep coordinates replication initiation using two distinct DNA recognition interfaces**. *Mol. Cell* 2004, **13**:403-414.

65. Pascal JM, O'Brien PJ, Tomkinson AE, Ellenberger T: **Human DNA ligase I completely encircles and partially unwinds nicked DNA**. *Nature* 2004, **432**:473-47810.1038/nature03082.

66. Brissett NC, Pitcher RS, Juarez R, Picher AJ, Green AJ, Dafforn TR, Fox GC, Blanco L, Doherty AJ: **Structure of a NHEJ polymerase-mediated DNA synaptic complex**. *Science* 2007, **318**:456-45910.1126/science.1145112.

67. Nandakumar J, Nair PA, Shuman S: **Last stop on the road to repair: structure of E. coli DNA ligase bound to nicked DNA-adenylate**. *Mol. Cell* 2007, **26**:257-27110.1016/j.molcel.2007.02.026.

68. Dürr H, Körner C, Müller M, Hickmann V, Hopfner K: **X-ray structures of the Sulfolobus solfataricus SWI2/SNF2 ATPase core and its complex with DNA**. *Cell* 2005, **121**:363-37310.1016/j.cell.2005.03.026.

69. Vanamee ES, Viadiu H, Kucera R, Dorner L, Picone S, Schildkraut I, Aggarwal AK: **A view of consecutive binding events from structures of tetrameric endonuclease SfiI**

bound to DNA. *EMBO J* 2005, **24**:4198-420810.1038/sj.emboj.7600880.

70. Kaus-Drobek M, Czapinska H, Sokołowska M, Tamulaitis G, Szczepanowski RH, Urbanke C, Siksnys V, Bochtler M: **Restriction endonuclease MvaI is a monomer that recognizes its target sequence asymmetrically**. *Nucleic Acids Res* 2007, **35**:2035-204610.1093/nar/gkm064.

71. Löwe J, Ellonen A, Allen MD, Atkinson C, Sherratt DJ, Grainge I: **Molecular mechanism of sequence-directed DNA loading and translocation by FtsK**. *Mol. Cell* 2008, **31**:498-50910.1016/j.molcel.2008.05.027.

72. Golovenko D, Manakova E, Tamulaitiene G, Grazulis S, Siksnys V: **Structural mechanisms for the 5'-CCWGG sequence recognition by the N- and C-terminal domains of EcoRII**. *Nucleic Acids Res* 2009, **37**:6613-662410.1093/nar/gkp699.

73. Lambert AR, Sussman D, Shen B, Maunus R, Nix J, Samuelson J, Xu S, Stoddard BL: **Structures of the rare-cutting restriction endonuclease NotI reveal a unique metal binding fold involved in DNA binding**. *Structure* 2008, **16**:558-56910.1016/j.str.2008.01.017.

74. Georgescu RE, Kim S, Yurieva O, Kuriyan J, Kong X, O'Donnell M: **Structure of a sliding clamp on DNA**. *Cell* 2008, **132**:43-5410.1016/j.cell.2007.11.045.
