# Supplementary material for: Re-visiting protein-centric two-tier classification of existing DNA-protein complexes
Source: BMC Bioinformatics. 2012 Jul 16;13:165. doi: 10.1186/1471-2105-13-165 (PMC3472317; doi:10.1186/1471-2105-13-165)
Supplement: Additional file 3 — Ternary protein-DNA complexes. [file 1471-2105-13-165-S3.pdf]

Additional file 3: Ternary DNA-protein complexes

Ternary Complex	Type I	Type II
1MDM	Homeodomain	Ets
	(HTH)	(wHTH)
1T2K	Interferon regulatory factor	bzip
	(wHTH)	(Zipper)
1GTO	Homeodomain	HMG
	(HTH)	(Other alpha)
1NGM	TF2b	Tata Box
	(HTH)	(Beta sheet)
1NH2	TF2b	Tata Box
	(HTH)	(Beta sheet)
1NVP	TF2b	Tata Box
	(HTH)	(Beta sheet)
1RM1	TF2b	Tata Box
	(HTH)	(Beta sheet)
2O61	Inteferon Regulatory	Ig fold like
	(wHTH)	(Other)
1H88	Rap1	bzip
	(HTH)	(zipper)
1H89	Rap1	bzip
	(HTH)	(zipper)
1H8A	Rap1	bzip
	(HTH)	(zipper)
2D5V	Homeodomain	Cut domain
	(HTH)	(Other alpha)
