# Supplementary figures and images for: Re-visiting protein-centric two-tier classification of existing DNA-protein complexes
Source: BMC Bioinformatics. 2012 Jul 16;13:165. doi: 10.1186/1471-2105-13-165 (PMC3472317; doi:10.1186/1471-2105-13-165)

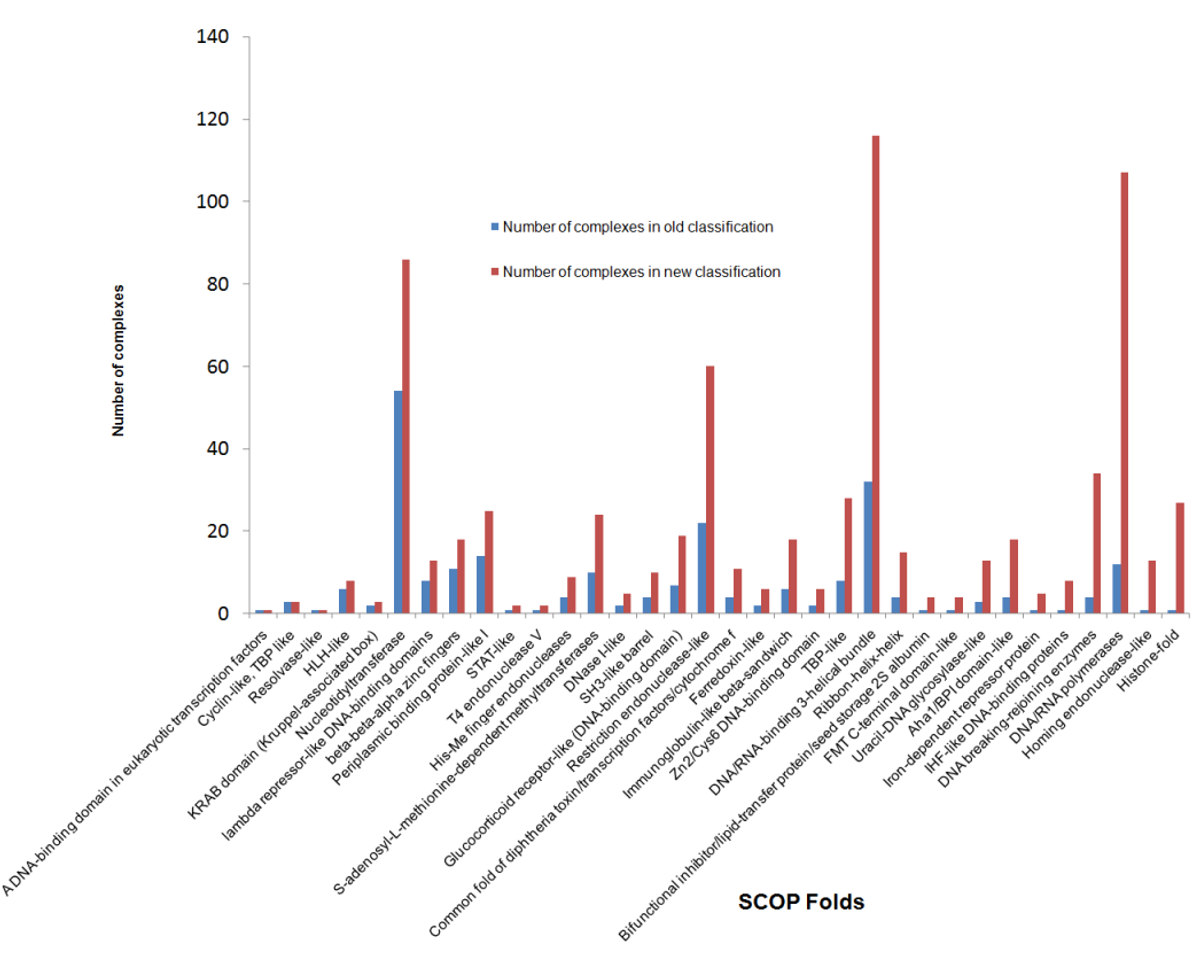

Supplement: Additional file 4 — The number of complexes in both old and new classification possessing each of the common 34 folds. [file 1471-2105-13-165-S4.tiff]

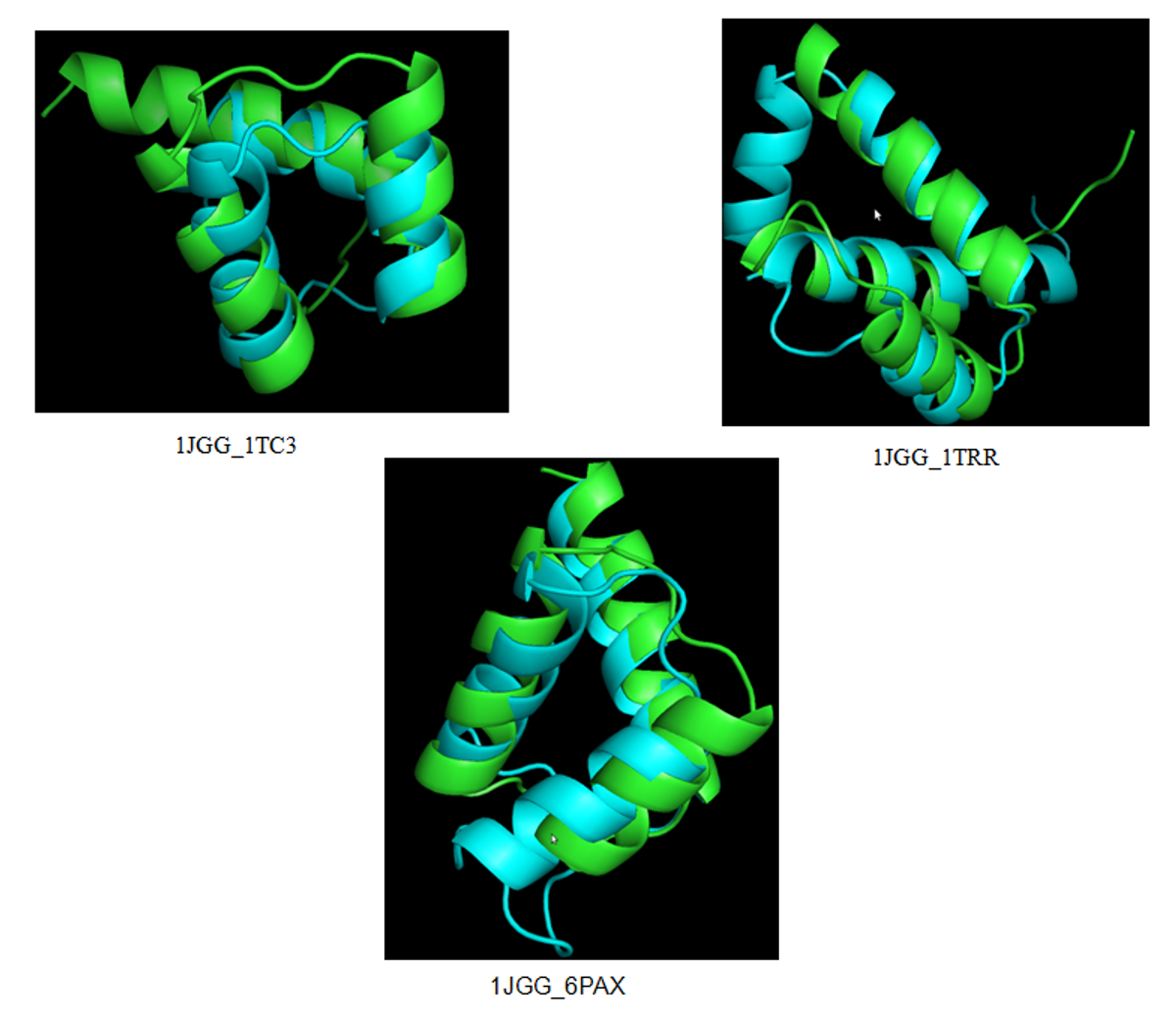

Supplement: Additional file 9 — Structural superposition using MATT. Structural alignment for 1JGG (Homeodomain new member) with 1TRR (Trp Repressor representative), 1TC3 (Tc3 transposase representative) and 6PAX (Homeodomain representative)]. [file 1471-2105-13-165-S9.tiff]
