# Supplementary material for: Re-visiting protein-centric two-tier classification of existing DNA-protein complexes
Source: BMC Bioinformatics. 2012 Jul 16;13:165. doi: 10.1186/1471-2105-13-165 (PMC3472317; doi:10.1186/1471-2105-13-165)
Supplement: Additional file 8 — RPS-BLAST alignment of new member of Homeodomain family (1JGG) with Homeodomain representatives (1HDD and 1FJL). [file 1471-2105-13-165-S8.pdf]

Additional file 8: RPS-BLAST alignment of new member of Homeodomain family (1JGG) with Homeodomain representatives (1HDD and 1FJL).

```
Query= 1JGG
                                                                Score    E
Sequences producing significant alignments:                     (bits) Value

1HDD                                                                  61   6e-13
1FJL                                                                  45   3e-08

>1HDD
          Length = 57

 Score = 60.5 bits (147), Expect = 6e-13
 Identities = 27/56 (48%), Positives = 42/56 (75%)

Query: 1   RYRTAFTRDQLGRLEKEFYKENYVSRPRRCELAAQLNLPESTIKVWFQNRRMKDKR 56
          R RTAF+ +QL RL++EF +  Y++  RR +L+++L L E+ IK+WFQN+R K K+
Sbjct: 1   RPRTAFSSEQLARLKREFNENRYLTERRRQQLSSELGLNEAQIKIWFQNKRAKIKK 56



 Score = 60.5 bits (147), Expect = 6e-13
 Identities = 27/56 (48%), Positives = 42/56 (75%)

Query: 58  RYRTAFTRDQLGRLEKEFYKENYVSRPRRCELAAQLNLPESTIKVWFQNRRMKDKR 113
           R RTAF+ +QL RL++EF +  Y++  RR +L+++L L E+ IK+WFQN+R K K+
Sbjct: 1   RPRTAFSSEQLARLKREFNENRYLTERRRQQLSSELGLNEAQIKIWFQNKRAKIKK 56


>1FJL
          Length = 123

 Score = 45.0 bits (107), Expect = 3e-08
 Identities = 45/108 (41%), Positives = 62/108 (57%), Gaps = 7/108 (6%)

Query: 13  RLEKEFYKENYVSRPRRCELAAQLNLPESTIKVWFQNRRMKDKRQ-------RYRTAFTR 65
            LE+ F +  Y     R ELA + NL E+ I+VWFQNRR + ++Q       R RT F+
Sbjct: 16  ELERAFERTQYPDIYTREELAQRTNLTEARIQVWFQNRRARLRKQHTSVSQRRSRTTFSA 75

Query: 66  DQLGRLEKEFYKENYVSRPRRCELAAQLNLPESTIKVWFQNRRMKDKR 113
            QL  LE+ F +  Y     R ELA + NL E+ I+VWFQNRR + ++
Sbjct: 76  SQLDELERAFERTQYPDIYTREELAQRTNLTEARIQVWFQNRRARLRK 123



 Score = 35.4 bits (82), Expect = 2e-05
 Identities = 24/54 (44%), Positives = 33/54 (61%)

Query: 3   RTAFTRDQLGRLEKEFYKENYVSRPRRCELAAQLNLPESTIKVWFQNRRMKDKR 56
           RT F+  QL  LE+ F +  Y     R ELA + NL E+ I+VWFQNRR + ++
Sbjct: 70  RTTFSASQLDELERAFERTQYPDIYTREELAQRTNLTEARIQVWFQNRRARLRK 123
```
