# Supplementary material for: Re-visiting protein-centric two-tier classification of existing DNA-protein complexes
Source: BMC Bioinformatics. 2012 Jul 16;13:165. doi: 10.1186/1471-2105-13-165 (PMC3472317; doi:10.1186/1471-2105-13-165)
Supplement: Additional file 10 — Unique hits obtained by profile-based methods. Some examples of DNA-binding proteins identified using only profile-based searches are observed during genome-wide survey in Arabidopsis thaliana. [file 1471-2105-13-165-S10.pdf]

Additional file 10: Examples of DNA-binding proteins identified by only profile based search methods RPS-BLAST and HMM in *Arabidopsis thaliana*

**Arabidopsis Protein ID**	**Arabidopsis Protein name**
AT1G01260.1	basic helix-loop-helix (bHLH) DNA-binding superfamily protein
AT1G07980.1	nuclear factor Y, subunit C10
AT1G08000.1	GATA transcription factor 10
AT1G01380.1	Homeodomain-like superfamily protein
AT3G29340.1	zinc finger (C2H2 type) family protein
